# Synthesis and Characterization of Glutamic-Chitosan Hydrogel for Copper and Nickel Removal from Wastewater

**DOI:** 10.3390/molecules21060684

**Published:** 2016-05-25

**Authors:** Huda E. Abdelwahab, Seham Y. Hassan, Mohamed A. Mostafa, Mohamed M. El Sadek

**Affiliations:** Chemistry Department, Faculty of Science, Alexandria University, Alexandria 21231, Egypt; Ehuda_eid@yahoo.com (H.E.A.); sehamyassen@yahoo.com (S.Y.H.); mabdelzaher15@yahoo.com (M.A.M.)

**Keywords:** chitosan, wastewater, copper(II), nickel(II), glutamic acid

## Abstract

Chitosan was reacted with four concentrations (2.5, 5, 10 and 20 mmol) of glutamic acid resulting in four types of glutamic-chitosan hydrogels (GCs), the activity of the resulted compounds on the removal of copper(II) and nickel(II) from wastewater were tested. The results indicated that by increasing glutamic acid concentration from GCs-1 to GCs-4, the efficiency of removing Cu(II) and Ni(II) were decreased, which may be due to a decrease in the pore size of the hydrogels as a result of the increased degree of crosslinking.

## 1. Introduction

The huge increase in the use of heavy metals over the past few decades has resulted in an unwanted increased presence of heavy metals in the environment, for example, industrial wastewater which contains high amount of heavy metals can pollute water resources. Heavy metals which include zinc, copper, nickel, mercury, cadmium, lead and chromium are one of the most toxic types of water pollutants. At least 20 metals are considered to be toxic and approximately half of these metals are emitted to the environment in quantities that are risky to the surroundings, in addition to the human health. A majority of heavy metals are non-biodegradable and highly toxic [1], so their concentrations have to be reduced to acceptable levels before discharge into the environment; otherwise, they can pose a threat to the health of animals and humans.

Wastewater containing heavy metals results mainly from metal plating facilities, mining operations, batteries, paper, fertilizer, tanneries, pesticide industries, stabilizers, thermoplastics, and pigment manufacture [2]. These industries discharge heavy metals directly or indirectly into the environment, especially in developing countries. Heavy metals tend to accumulate in living organisms [3] causing numerous diseases and disorders due to their toxicity and non-biodegradability [4]. Nickel is a chemically active metal used for preparing a large number of nickel alloys and also used in many other industries. The excessive intake of nickel may cause carcinogenesis, mutagenesis and dermatogenic effects as a result of bioaccumulation [5,6]. Copper (Cu(II)) is micronutrient element that plays an important role in bone formation together with certain proteins and enzymes [7]. However, the consumption of food or water containing high copper concentrations can cause several diseases such as gastrointestinal symptoms, liver toxicity, osteoporosis, Wilson’s, and Alzheimer’s diseases [8,9,10]. Excessive intake of copper can also cause hemolysis, hepatotoxicity, nephrotoxicity, vomiting, cramps, and convulsions [4].

The high cost and complexity, high energy consumption and secondary pollution problems [11] of most of the treatment processes that are used to remove heavy metals from wastewater are considered to be major problems [12]; for those reasons, a number of studies were carried out on the use of low-cost adsorbents for the removal of heavy metals from natural resources [13] such as chitosan, which also is a biodegradable and biocompatible polymer. Chitosan consists mainly of β-(1→4)-2-acetamido-2-deoxy-d-glucose units which are produced by deacetylation of chitin [14]. Chitosan is the second most abundant biopolymer on Earth after cellulose [15], it is widely distributed in crustacean shells and cell walls of fungus. Several methods have been reported for the chemical modification of chitosan, one of which is the crosslinking of chitosan with various substances such as dialdehydes and dicarboxylic acids. Chitosan was modified with several dicarboxylic acids including glutamic acid by reacting both carboxylic acid groups with the amino groups of chitosan [6]. In the present work, chitosan was reacted with four different amounts of glutamic acid, resulting in four types of glutamic-chitosan (GC) hydrogels, the resulting crosslinking polymers were tested for the removal of copper(II) and nickel(II) from wastewater.

## 2. Results and Discussion

### 2.1. Synthesis of Glutamic-Chitosan Cross Linked Hydrogels (GCs)

**Scheme 1:**
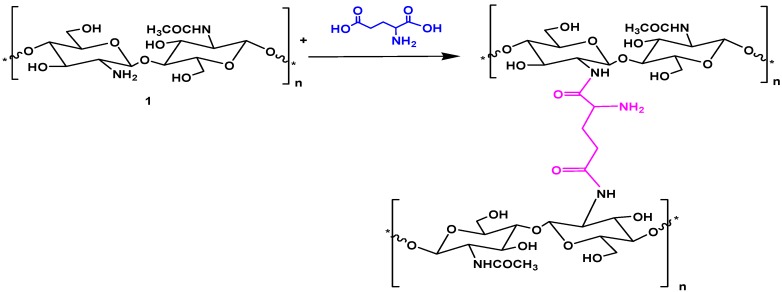
Synthesis of glutamic-chitosan hydrogel.

Chitosan was modified with glutamic acid, whereby the carboxylate groups of glutamic acid reacted with the amino groups of chitosan (Scheme 1). The amount of glutamic acid with respect to chitosan was varied to produce four new cross-linked glutamic-chitosan hydrogels (Scheme 1) designated as: glutamic-chitosan-1 (GCs-1), glutamic-chitosan-2 (GCs-2), glutamic-chitosan-3 (GCs-3), and glutamic-chitosan-4 (GCs-4) with increasing degrees of cross linking, respectively. All the prepared derivatives are produced in a nearly quantitative yield (89%–96.4%).

**Scheme 2:**
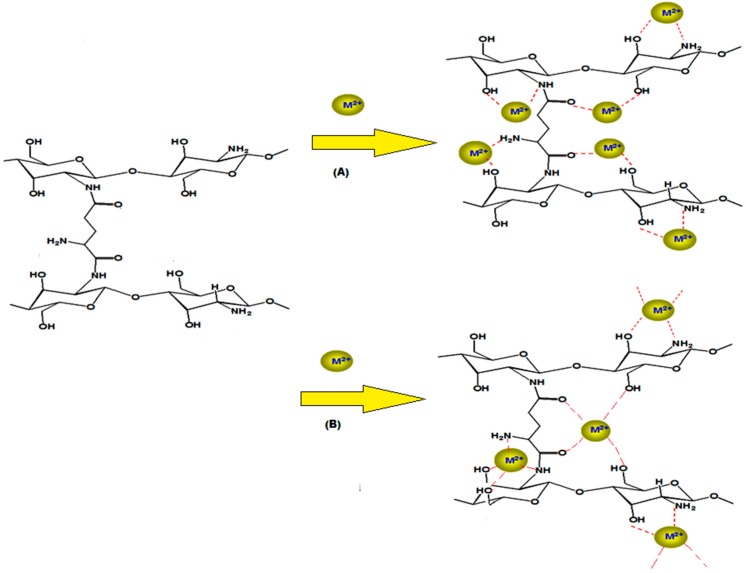
The proposed mechanism, M^2+^ for (**A**) nickel and (**B**) copper.

The modification of chitosan with crosslinking reactions leads to formation of chitosan derivatives with better resistance in extreme media conditions [16]. On the other hand, the crosslinking modification slightly decreases the adsorption capacity of chitosan; to overcome this difficulty we choose glutamic acid as crosslinker since glutamic acid will increase the amino (-NH_2_) and the carbonyl (C=O) groups [17]. Increasing the abundance of these groups in the target molecule will facilitate increased complex formation with Ni(II) and Cu(II). The proposed adsorption mechanism of Ni(II) and Cu(II) on chitosan-glutamic hydrogel is illustrated in Scheme 2.

### 2.2. Fourier Transform Infrared Spectroscopy (FTIR) Characterization of G-Cs

Glutamic-chitosan formation was confirmed using Fourier transform infrared spectroscopy. The FTIR spectrum of chitosan showed four strong absorption peaks at 1157.8, 1076.6, 1030, and 895.7 cm^−1^ which are characteristic peaks of the saccharide structure, where the OH and NH functions showed a very strong broad absorption peak around 3600–3200 cm^−1^. Primary amines showed two absorption peaks at 1650.4 and 1598.9 cm^−1^, which indicated that chitosan had a high degree of deacetylation [18].

The FTIR spectra of the GC hydrogels showed the broad band between 3450 and 3470 cm^−1^ due to the OH and NH groups. In addition, the characteristic absorbance of NH_2_ at 1650.4 and 1598.9 cm^−1^ was also seen. The spectra also showed a broad absorption band around 1637 cm^−1^ which corresponds to the (CONH) amide group, and the intensity of this band increased with increasing cross-linking density of the hydrogels, *i.e.*, from GCs-1 to GCs-4 (see Table 1).

### 2.3. Elemental Characterization of GCs

Elemental analyses of the GC derivatives is another confirmation of GC formation; the elemental analysis data are shown in Table 2.

### 2.4. ^1^H-NMR Characterization of GC Hydrogels

The structure of compounds GCs-1 to GCs-4 is further proved by ^1^H-NMR spectroscopy, which showed the two (NH) protons as a singlet at δ 7.77, the 1′-OH proton at 4.00, and the rest of the sugar protons at the range 3.29–3.33 ppm, as well as the appearance of the two (NH_2_) protons f at 3.29 (see Experimental). After shaking of compounds **1**–**4** with D_2_O, their ^1^H-NMR spectra showed the disappearance of the (NH_2_) and (NH) protons as well as (OH) protons [18].

### 2.5. Scanning Electron Microscopy Observations of G-Chitosan Hydrogels

**Figure 1:**
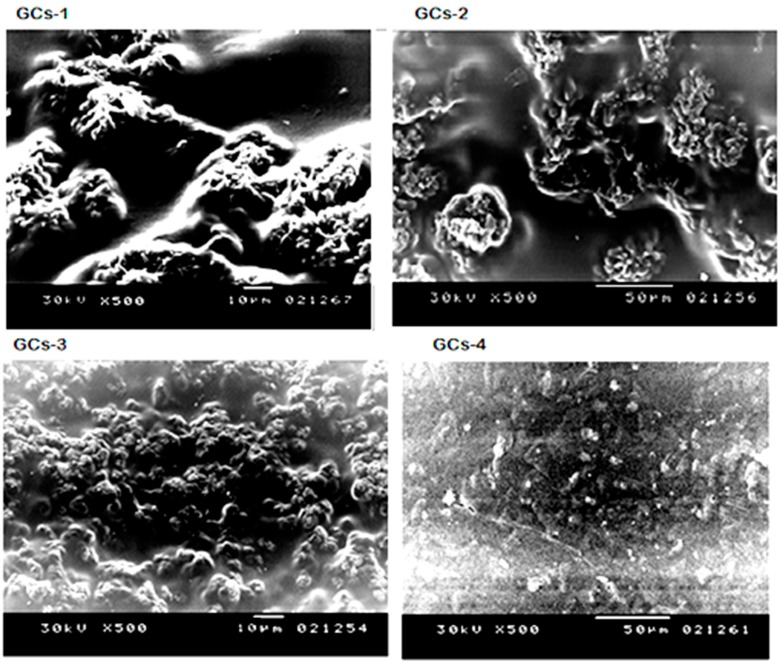
Scanning electron microscopy of G-chitosan hydrogels.

Microstructures of the hydrogels surface were investigated by scanning electron microscopy as presented in Figure 1. It could be seen that the hydrogels have a similar surface appearance, but the distribution and the size of their pores are different. The porosity distribution became more uniform and dense with increasing concentration of glutamic acid.

The extremely porous surface structure of the hydrogels could lead to high surface areas. The pore size of the hydrogels decreased with increasing the cross-linking density of the hydrogels from GCs-1 to GCs-4 hydrogel.

### 2.6. Solubility of G-Chitosan Hydrogels

The solubility of the new hydrogels was studied in different solvents at room temperature. The results show that the hydrogels are insoluble in acetic acid solution (1% *v*/*v*), dimethylformamide, dimethyl sulfoxide, tetrahydrofuran, *N*-methylpyrrolidone, chloroform, methylene chloride, acetone and methanol since no soluble fractions of the hydrogels were obtained. This indicates a successful formation of crosslinked networks in these hydrogels.

### 2.7. Sorption Studies of Ni(II) and Cu(II)

#### 2.7.1. Influence of G-chitosan Amount

**Figure 2:**
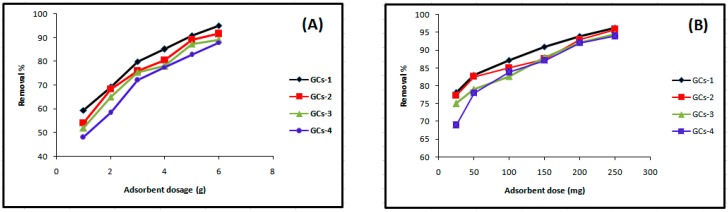
Influence of GCs amount on (**A**) nickel and (**B**) copper sorption.

The dependence of Ni(II) sorption on G-chitosan amount was studied by varying the amount of the adsorbent from 1 g to 5 g while keeping the other parameters such as pH, metal solution volume (100 mL), concentration (200 mg/L), and contact time (60 min) constant. Figure 2A shows that the percentage removal of nickel increases with increasing adsorbent dose from 48% to 95%.

Figure 2B shows that the removal efficiency of copper was improved on increasing adsorbent doses; this may occur due to the fact that the higher dose of adsorbents in the solution provides the greater availability of exchangeable sites for the ions. The maximum % removal of Cu(II) was 95.17% at the dosage of 250 mg.

#### 2.7.2. Influence of pH

**Figure 3:**
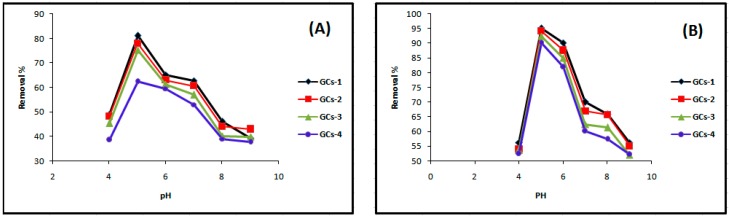
Influence of pH on (**A**) nickel and (**B**) copper sorption.

The effect of pH on the adsorption of Ni is presented in Figure 3A. The pH of the aqueous solution is an important parameter in the adsorption process because it affects the concentration of the counter ions on the functional groups of the adsorbent, the solubility of the metal ions and the degree of ionization of the adsorbate during reaction [12]. The active sites on an adsorbent can either be protonated or deprotonated depending on the pH while at the same time the adsorbate speciation in solution depends on the pH too.

**Figure 4:**
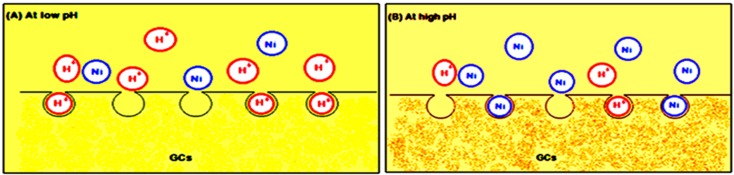
Metal ion uptake (**A**) at low pH and (**B**) at high pH.

At low pH (2–4), less metal ion uptake was observed due to the competitive adsorption of the H^+^ and Ni(II) ions on the G-chitosan compounds surface. At low pH values, the adsorbent is positively charged with higher H^+^ ion concentration, reducing the number of binding sites for metal ion (Figure 4). In addition, the protonation of amino groups in acidic solution induces an electrostatic repulsion of metal cations that reduces the number of binding sites available for metallic ions [13]. However, Ni(II) uptake increased as the pH increased to pH 9, as most active sites on the adsorbent are deprotonated resulting to a more net attractive force which is responsible for high nickel removal from aqueous solution. The optimum adsorption takes place at pH 5. Further increase in pH leads to the precipitation of nickel hydroxide complexes which inhibits the adsorption process.

Figure 3B as shown above illustrated that pH obviously influenced the removal efficiency of the copper ions in the aqueous solution; the results indicated that Cu(II) removal was increased to maximum and then decreased with pH variation from 4 to 9 at 25°C and agitation speed of 100 rpm. The maximum % removal of Cu(II) was 95% at pH 5. The dominant species of copper was free Cu(II) and was mainly involved in the adsorption process when the pH was lower than 5. With the pH greater than 5, copper ions started to precipitate as Cu (OH) [13]. Increases in metal removal with increased pH can be explained on the basis of the decrease in competition between proton and metal cations, which results in a lower electrostatic repulsion between surface and metal ions. Decrease in adsorption at higher pH (>5) is due to the formation of soluble hydroxyl complexes [15].

#### 2.7.3. Influence of Contact Time

**Figure 5:**
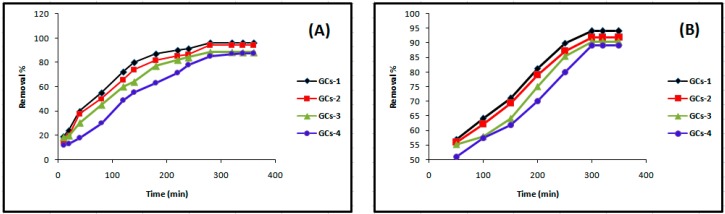
Influence of contact time on (**A**) nickel and (**B**) copper sorption.

Contact time is one of the effective factors in the batch adsorption process. Keeping other parameters including temperature (25 °C), pH 5, adsorbent dose (1 g/100 mL), initial nickel concentration (200 mg/L) and agitation speed (250 rpm) constant, the adsorption of nickel on glutamic-chitosan compounds was studied in the range 10–360 min. The effect of contact time on nickel adsorption efficiency is shown in Figure 5A. Adsorption rate initially increased rapidly, and the optimal removal efficiency was reached within 280 min. No change in nickel concentration after 280–360 min was observed. The availability of sufficient vacant adsorbing sites in the beginning of the removal process is possibly the cause of the higher initial removal; afterwards, the removal percent rate decreased due to the limited vacant adsorption sites.

Figure 5B indicates that Cu(II) removal was increased from 50% to 92% as the contact time varied from 10 min to 360 min. The optimum contact time for maximum removal (92%) of Cu(II) was 300 min.

#### 2.7.4. Influence of Initial Concentration

**Figure 6:**
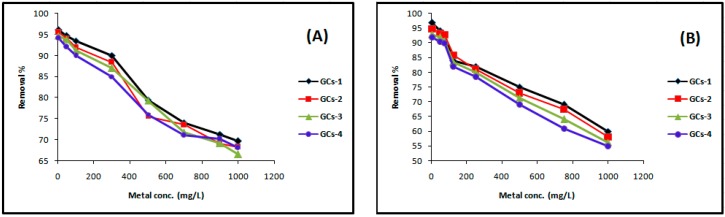
Influence of the initial concentration on (**A**) nickel and (**B**) copper sorption.

The experimental results of the effect of initial nickel concentration on removal efficiency are presented in Figure 6A. The experiment was conducted using the volumes of solutions as 100 mL, initial concentrations of metal as 5, 50, 100, 300, 500, 700, 900 and 1000 mg/L solution of Ni(II) in conical flasks, were gently shaken with 1 g of G-chitosan compounds for 60 min with 250 rpm in the orbit mechanical shaker, with initial pH of the solution 5. Figure 6A shows the nickel removal efficiency decreased with the increase in initial nickel concentration. In the case of low nickel concentrations, the ratio of the initial number of moles of nickel ions to the available surface area of adsorbent is large. However, at higher concentrations, the available sites of adsorption become fewer and hence the percentage removal of metal ions which depends on the initial concentration decreases [2]. The removal percentage decreases from 96% to 66% as the concentration increases.

The experimental results of the effect of initial copper concentration on removal efficiency were presented in Figure 6B, which showing that the copper removal efficiency decreased with the increase in initial copper concentration [19]. The removal percentage decreases from 92%–55%, 93%–56%, 95%–58% and 97%–60% as the concentration increases.

### 2.8. Sorption Isotherm Studies

#### 2.8.1. Langmuir Sorption Isotherm

The maximum amount of Ni(II) or Cu(II) ion adsorption on the modified polymer is defined by the corresponding adsorption isotherms [17]. The Langmuir isotherm is based on the assumption that the adsorbent surface has sites with identical energy and has equal affinities for the adsorbate molecules, which mean that each adsorbate molecule is assumed to be located on a single site. Langmuir model predicts the formation of the monolayer of the adsorbate [20]. The experimental adsorption data are fitted according to the Langmuir isotherm models, from the equation:

*C_e_*/*Q_e_* = *C_e_*/*Q_m_* + 1/(*K_L_* × *Q_m_*) (1)

where *C_e_* is the equilibrium concentration of the adsorbate (mg/L), *Q_m_* is the maximum adsorption capacity of the adsorbent and *K_L_* is the Langmuir adsorption constant related to capacity and energy of adsorption, respectively; *Q_e_* is the adsorption quantity (mg/g) which calculated by the following equation:

*Q_e_* = [(*C*_0_ – *C*)*V*]/*W* (2)

where *C*_0_ is the initial Ni(II) or Cu(II) concentration (mg/L), *C* is final concentration after the adsorption; *V* is the solution volume (L); and *W* is the weight of the used adsorbent (g).

**Figure 7:**
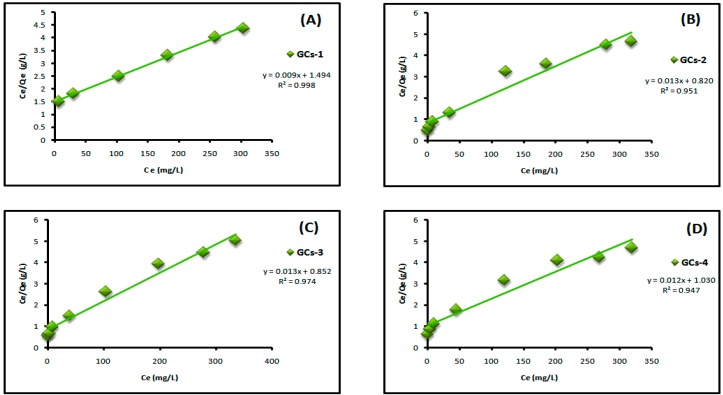
Langmuir isotherm for Ni(II) adsorption using (**A**) GCs-1; (**B**) GCs-2; (**C**) GCs-3 and (**D**) GCs-4.

**Figure 8:**
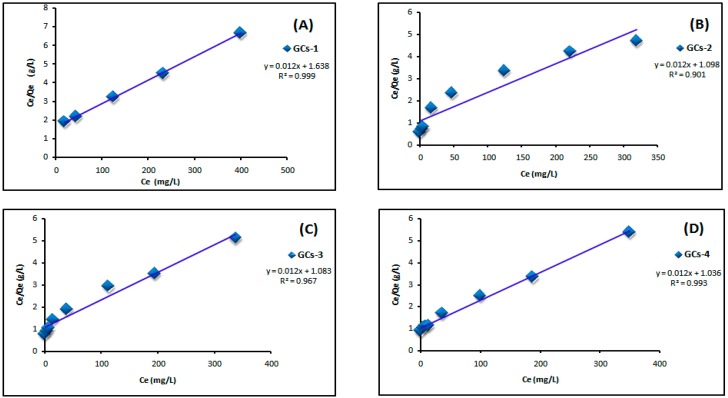
Langmuir isotherm for Cu(II) adsorption using (**A**) GCs-1; (**B**) GCs-2; (**C**) GCs-3 and (**D**) GCs-4.

Figure 7 shows the Langmuir adsorption isotherm of adsorption of nickel ions on chitosan derivatives GCs-1, GCs-2, GCs-3 and GCs-4 using Ni(II) concentrations (100–1300 mg/L) at pH 5 and 1 g of adsorbent, where Figure 8 shows the Langmuir adsorption isotherm of adsorption of copper ions on chitosan derivatives GCs-1, GCs-2, GCs-3 and GCs-4 using Cu(II) concentrations (75–1300 mg/L) at pH 5 and 1 g of adsorbent.

By plotting *C_e_*/*Q_e_* *versus C_e_* as shown in Figure 7 and Figure 8 for nickel and copper, respectively; it was found that the experimental adsorption data are fitted according to the Langmuir isotherm models with correlation coefficient values *R^2^* = (0.998, 0.951, 0.974, 0.947) and (0.999, 0.901, 0.967, 0.993) for Ni(II) and Cu(II), respectively. Both *Q_m_* and *K_L_* can be calculated from the slope and the intercept of the linear plot in which; slope = 1/*Q_m_* and intercept = 1/*Q_m_* × *K_L_*. The fitting result showed that the maximum sorption capacity *Q_m_* of polymer adsorbent reached 103.4 mg/g in case of Ni(II) and 83.33 mg/g in case of Cu(II), these results confirm the applicability of Langmuir model which suggests that the adsorption was taken place as mono layer adsorption.

#### 2.8.2. Freundlich Sorption Isotherm

**Figure 9:**
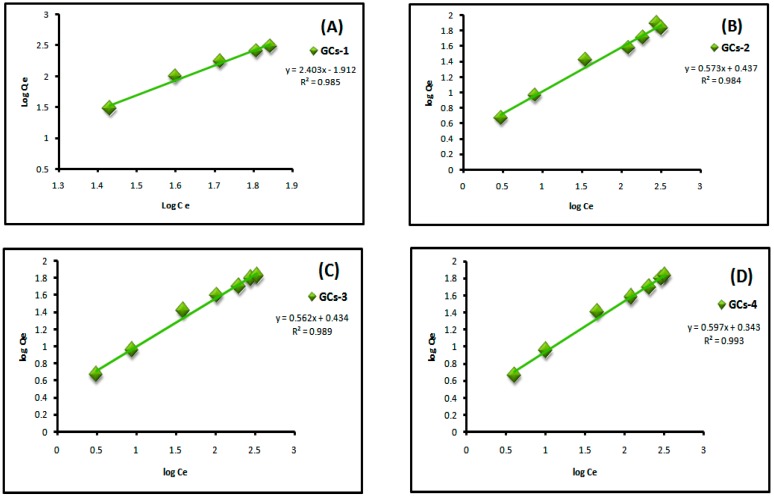
Freundlich plot for Ni(II) adsorption on to (**A**) GCs-1; (**B**) GCs-2; (**C**) GCs-3 and (**D**) GCs-4.

**Figure 10:**
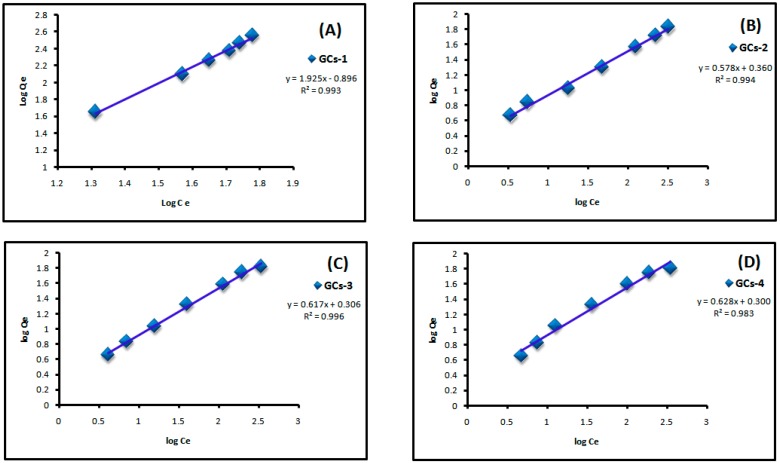
Freundlich plot for Cu(II) adsorption on to (**A**) GCs-1; (**B**) GCs-2; (**C**) GCs-3 and (**D**) GCs-4.

Surface heterogenty of the sorbent is indicated from Freundlich model which represented by the following equation:

log *Q_e_* = log *K_f_* + 1/*n* × log *C_e_* (3)

where *Q_e_* is the adsorbed amount at equilibrium (mg/g), *C_e_* is the equilibrium concentration of Cu(II) or Ni(II) (mg/L), *K_f_* is Freundlich constant, 1/*n* is Freundlich exponent. Both constants were calculated from the slope and intercept of the plotting between log *Q_e_* and log *C_e_*. Linear plot of log *C_e_ vs.* log *Q_e_* confirm the applicability of Freundlich model as shown in Figure 9 and Figure 10. It means that the adsorption was taken place at a heterogeneous surface; however nickel and copper adsorption on glutamic-chitosan are fitted to both models since the correlation coefficients values are very close.

### 2.9. Kinetics Studies

The kinetic parameters for the adsorption process were studied for contact time from 10 min to 360 min by monitoring separately the percentages of removal of the Cu(II) and Ni(II). Pseudo-first order kinetics are represented by Equation (4), while the pseudo-second order ones are represented by Equation (5) [20]:

ln (*Q_e_* − *Q_t_*) = −*k*_1_*t + * ln *Q_e_* (4)

*t*/*Q_t_* = *1*/*k*_2_ × *Q_e_*^2^ × *t + t/Q_e_* (5)

where *Q_e_* and *Q_t_*: are the amount of metal adsorbed (mg/g) at equilibrium and at time *t* (min), *k*_1_ (min^−1^) and *k*_2_ (g·mg^−1^·min^−1^) are the adsorption rate constant of pseudo-first order, pseudo-second order adsorption kinetics, respectively. The values of *k*_1_ can be determined from the slope of the linear plot of ln *(Q_e_* − *Q_t_) vs. t*, and *k*_2_ can be calculated from the slope of the linear plot of *t*/*Q_t_ vs. t*.

**Figure 11:**
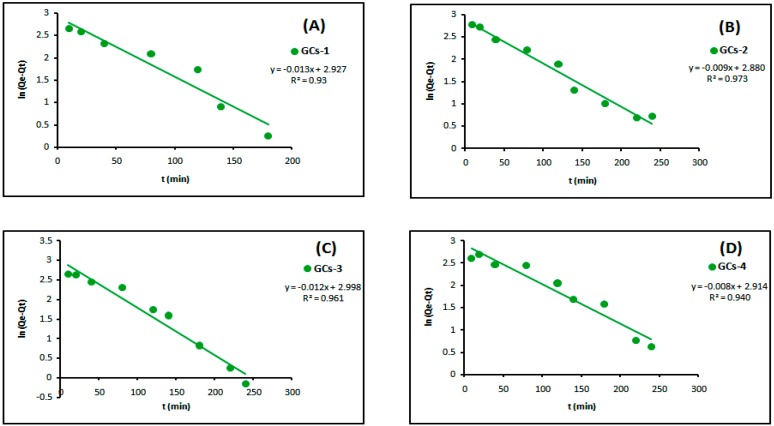
Pseudo-first order kinetics models of nickel adsorption using (**A**) GCs-1; (**B**) GCs-2; (**C**) GCs-3 and (**D**) GCs-4.

**Figure 12:**
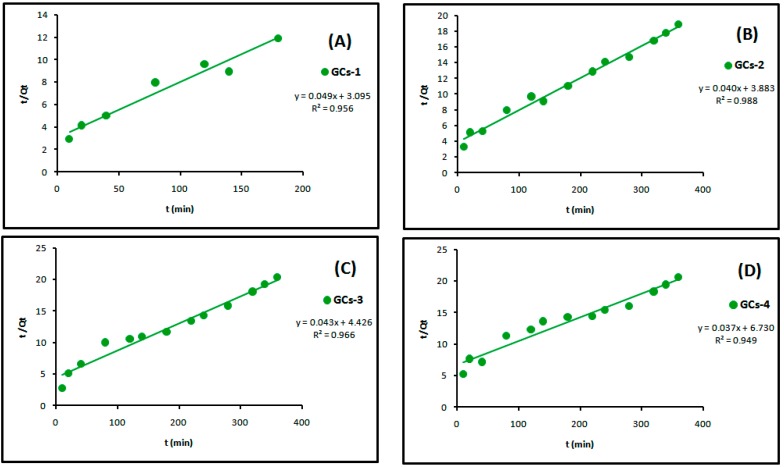
Pseudo-second order kinetics models of nickel adsorption using (**A**) GCs-1; (**B**) GCs-2; (**C**) GCs-3 and (**D**) GCs-4.

The linear plots of the two kinetic models of Ni(II) are presented in Figure 11 and Figure 12, respectively. Table 3 showed the values of *k*_1_, *k*_2_, *Q_e_* and the correlation coefficient (*R*^2^) from the linear plots. The pseudo-second order linear plots of Ni(II) resulted in higher *R*^2^ values than the pseudo-first order. These indicated better applicability of the pseudo-second order model, which relies on the assumption that chemisorptions are the rate limiting step.

**Figure 13:**
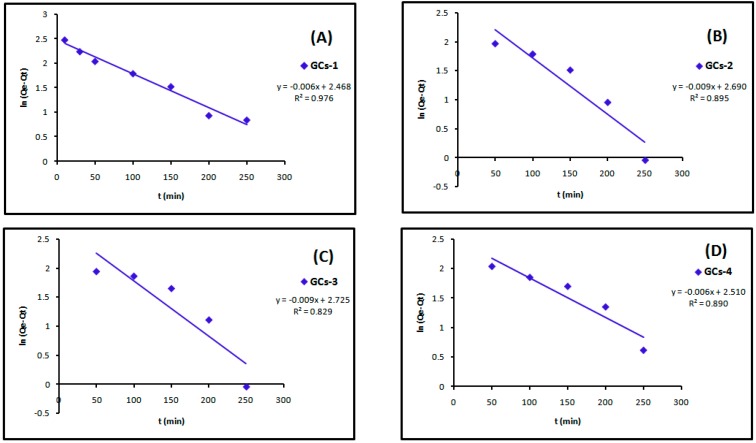
Pseudo-first order kinetics models of copper adsorption using (**A**) GCs-1; (**B**) GCs-2; (**C**) GCs-3 and (**D**) GCs-4.

**Figure 14:**
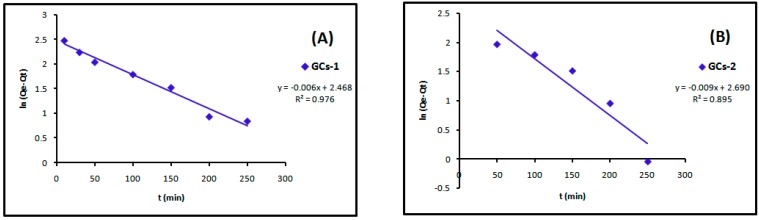

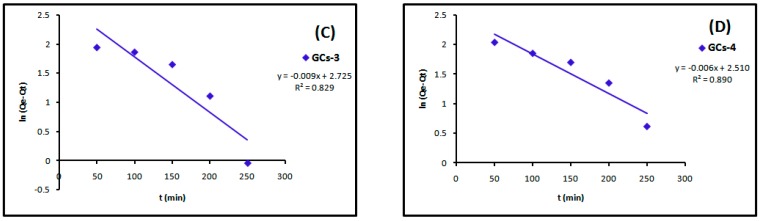
Pseudo-second order kinetics models of copper adsorption using (**A**) GCs-1; (**B**) GCs-2; (**C**) GCs-3 and (**D**) GCs-4.

The linear plots of the two kinetic models of Cu(II) are presented in Figure 13 and Figure 14. Table 4 showed the values of *k*_1_, *k*_2_, *Q_e_* and the correlation coefficient (*R*^2^) from the linear plots. The pseudo-second order linear plots of Cu(II) resulted in higher *R*^2^ values than the pseudo-first order.

### 2.10. Desorption Studies of Ni(II) and Cu(II)

The sorbed Ni and Cu were dried and preserved. For desorption Cu and Ni-sorbed chitosan were shaken with dilute hydrochloric acid (0.5 M) for 1 h at room temperature and filtered. The filtrate was analyzed for the oxidation states of Cu and Ni using UV-Visible spectrophotometry in the wavelength ranges 190 nm–1100 nm.

## 3. Materials and Methods

### 3.1. Materials

Chitosan was purchased from Acros Organics (Morris Plains, NJ, USA). Its deacetylation degree is 88% and its average molecular weight is 100,000–300,000 Da. Glutamic acid, acetic acid, and methanol, were of analytical grade from Aldrich (Saint Louis, MO, USA) and used as received.

### 3.2. Measurements

Fourier transform infrared (FTIR) spectra were recorded using KBr discs on a Perkin Elmer spectrometer (Perkin-Elmer, Waltham, MA, USA) at room temperature in the range of 4000–400 cm^−1^. Elemental analyses of the prepared derivatives were done in a Model 2410-Series II C, H, N, S Analyzer (Perkin-Elmer, Shelton, CT, USA). ^1^H-NMR spectra were recorded using a Gemini-300 MHz instrument (Gemini, Palo Alto, CA, USA), in DMSO-*d*_6_ as a solvent at 25 °C. Chemical shifts (δ) are expressed in parts per million (ppm) from tetramethylsilane as an internal standard. The morphology of the cross-linking gel was analyzed with a JEOL-JSM 5300 Scanning electron microscope (JEOL, Tokyo, Japan); metal solutions were analyzed using a Model U-2800 UV-Visible spectrophotometer (Hitachi, Schaumburg, IL, USA). Ni(II) and Cu(II) concentrations were measured using a Thermo-Scientific ICE-3300 Atomic adsorption spectrometer AAs (Thermo-Scientific, Waltham, MA, USA).

### 3.3. General Procedures for Chitosan-Glutamic Synthesis

A solution of glutamic acid in distilled water (20 mL) was added to chitosan (20 mmol) in distilled water (30 mL). The mixture was stirred for 4 h at 60 °C. After cooling, the homogenous cross-linked hydrogels which formed was submerged in methanol for 12 h for dewatering to give white product; the dewatering hydrogel was filtered and dried at 60 °C to constant weight.

G-chitosan-1: Obtained from glutamic acid (2.5 mmol) in 96.4% yield; IR (KBr): 1568 (CONH), 1631 (CONH), 2867, 2925 (NH_2_), 3437 cm^−1^ (NH), (OH); ^1^H-NMR (δ, ppm) (DMSO-*d*_6_) δ: 1.88 (q, 2H, CH_2_-glutamic), 2.11 (m, 1H, H-5′), 2.29 (m, 2H, H-1′, H-2′), 2.35 (t, 2H, CH_2_CO), 2.60 (m, 2H, H-3′, H-4′), 2.61 (m, 2H, H-6a′, H-6b′), 3.27 (t, 1H, CH-CO), 3.29 (bs, NH_2_, OH’s; exchangeable with D_2_O), 4.00 (m, 1H, OH; exchangeable with D_2_O), 7.77 (s, 2H, 2NH; exchangeable with D_2_O). Anal. Found: C, 47.80; H, 7.15; N, 7.69.

G-chitosan-2: Obtained from glutamic acid (5 mmol) in 92.5% yield; IR (KBr): 1638 (CONH), (NH_2_), 3445 cm^−1^ (NH), (OH); Anal. Found: C, 47.84; H, 7.24; N, 7.72.

G-chitosan-3: Obtained from glutamic acid (10 mmol) in 90.2% yield; IR (KBr): 1636 (CONH), 2925 (saturated C-H), (NH_2_), 3728, 3434 cm^−1^ (NH), (OH); Anal. Found: C, 47.86; H, 7.27; N, 7.74

G-chitosan-4: Obtained from glutamic acid (20 mmol) in 89% yield; IR (KBr): 1637 (CONH), 2925 (saturated C-H), NH_2_, 3438 cm^−1^ (NH), (OH); Anal. Found: C, 47.88; H, 7.35; N, 7.77.

### 3.4. Stock Solution Preparation

Stock solution of 10 mg/L Cu(II) ion was prepared by dissolving copper sulphate pentahydrate (CuSO_4_·5H_2_O, 39.28 mg)in distilled water (1000 mL)contained in a volumetric flask. Ni(II) stock solution of 10 mg/L concentration was also prepared by dissolving nickel sulphate hexahydrate (NiSO_4_·6H_2_O, 43.96 mg) in 1000 mL distilled water. Hydrochloric acid and sodium hydroxide were used to adjust the solution pH. Distilled water was used throughout the experimental work.

### 3.5. Adsorption Experiments

Sorption experiments were conducted in 250 mL conical flasks containing 100 mL of various concentrations of Ni(II) or Cu(II) solution using accurately weighed chitosan. The flasks were agitated in an orbit shaker at 100 rpm and at room temperature (25 °C). The initial and final concentrations of the solutions were measured by atomic adsorption (AAS) at the maximum adsorption wavelength and the adsorption capacities of the adsorbent were calculated.

The percent removal of metals from the solution was calculated by the following equation [20]:

(6) $$\%\;\mathrm{removal}=\frac{C_{0}-C}{C_{0}}\times 100$$

where *C*_0_ (mg/L) is the initial metal ion concentration and *C* (mg/L) is the final metal ion concentration in the solution.

The effect of sorbent dosage was studied from 1 g to 5 g for 1 h contact time. Effect of initial pH on the sorption capacity of sorbent for Ni(II) and Cu(II) was studied by varying solution pH from 3 to 9 at the sorbent dosage of 1 g/100 mL for 1 h contact time using 200 mg/L initial Ni and Cu concentration; the solution pH was adjusted with dilute HCl or NaOH solution. The effect of contact time on the sorption capacity of sorbent was studied in the range 25–350 min at an initial Ni and Cu concentration of 200 mg/L. The effect of initial concentration on the sorption capacity of sorbent was studied using the volume of solutions as 100 mL, and initial concentration of nickel as 5, 50, 100, 300, 500, 700, 900 and 1000 mg/L; and copper as 5, 50, 75, 125, 250, 500, 750 and 1000 mg/L. Adsorption isotherms were studied at different initial Ni, Cu concentrations at room temperature.

## 4. Conclusions

Chitosan-glutamic hydrogels were synthesized by modification of chitosan with glutamic acid which was characterized with FTIR, ^1^H-NMR, elemental analysis and SEM. The influence of adsorbent dosage, solution pH value, reaction time and initial concentration of Ni(II) and Cu(II) on the adsorption capacity were investigated. The results indicate that chitosan-glutamic derivatives are good adsorbents of Ni(II) and Cu(II).

## Figures and Tables

**Table 1:** IR spectral data of compounds **1**–**4**.

Compound	IR (γ, cm^−1^)
GCs-1	1635 (CONH), 3465 (NH), 3465 cm^−1^ (OH)
GCs-2	1637 (CONH), 3458 (NH), 3458 cm^−1^ (OH)
GCs-3	1637 (CONH), 3467 (NH), 3467 cm^−1^ (OH)
GCs-4	1638 (CONH), 3467 (NH), 3467 cm^−1^ (OH)

**Table 2:** Elemental analyses and % yield of G-chitosan hydrogels.

Compound	Elemental Analyses			Yield %
	% C	% H	% N	
Cs	45.10	6.77	8.43	-
GCs-1	47.80	7.15	7.69	96.4
GCs-2	47.84	7.24	7.72	92.5
GCs-3	47.86	7.27	7.74	90.2
GCs-4	47.88	7.35	7.77	89

**Table 3:** Constants and correlation coefficient of pseudo-first order and pseudo-second order kinetics of nickel adsorption.

Metal Ion	Pseudo-First Order Model			Experimental Value	Pseudo-Second Order Model		
	*Q_e_* (mg/g)	*k*_1_ (min^−1^)	*R*^2^	*Q_e_* (mg/g)	*Q_e_* (mg/g)	*k*_2_ (g·mg^−1^·min^−1^)	*R*^2^
GCs-1	20.32	0.013	0.930	18.08	18.67	0.005	0.956
GCs-2	25.00	0.009	0.973	19.06	17.81	0.004	0.988
GCs-3	23.26	0.012	0.961	17.68	20.05	0.004	0.966
GCs-4	27.03	0.008	0.940	17.46	18.43	0.002	0.949

**Table 4:** Constants and correlation coefficient of pseudo-first order and pseudo-second order kinetics of copper adsorption.

Metal Ion	Pseudo-First Order Model			Experimental Value	Pseudo-Second Order Model		
	*Q_e_* (mg/g)	*k*_1_ (min^−1^)	*R*^2^	*Q_e_* (mg/g)	*Q_e_* (mg/g)	*k*_2_ (g·mg^−1^min^−1^)	*R*^2^
GCs-1	12.99	0.007	0.974	18.8	19.01	0.009	0.989
GCs-2	14.73	0.009	0.895	18.4	21.20	0.006	0.975
GCs-3	15.26	0.009	0.829	18.06	20.73	0.005	0.963
GCs-4	12.31	0.005	0.890	17.84	21.30	0.004	0.954
